# Supervised Molecular Dynamics (SuMD) Insights into the mechanism of action of SARS-CoV-2 main protease inhibitor PF-07321332

**DOI:** 10.1080/14756366.2021.1954919

**Published:** 2021-07-21

**Authors:** Matteo Pavan, Giovanni Bolcato, Davide Bassani, Mattia Sturlese, Stefano Moro

**Affiliations:** Molecular Modeling Section (MMS), Department of Pharmaceutical and Pharmacological Sciences, University of Padova, Padova, Italy

**Keywords:** SuMD, PF-07321332, SARS-CoV-2, Covid-19

## Abstract

The chemical structure of PF-07321332, the first orally available Covid-19 clinical candidate, has recently been revealed by Pfizer. No information has been provided about the interaction pattern between PF-07321332 and its biomolecular counterpart, the SARS-CoV-2 main protease (M^pro^). In the present work, we exploited Supervised Molecular Dynamics (SuMD) simulations to elucidate the key features that characterise the interaction between this drug candidate and the protease, emphasising similarities and differences with other structurally related inhibitors such as Boceprevir and PF-07304814. The structural insights provided by SuMD will hopefully be able to inspire the rational discovery of other potent and selective protease inhibitors.

## Introduction

The Covid-19 pandemic, caused by a single-stranded RNA betacoronavirus known as SARS-CoV-2, has caused the death of more than 3 million people around the world since its outbreak in December 2019^1^^,^^2^. Despite the impressive cooperative effort promoted by the international community and by medicinal chemists around the world^3^^,^^4^, to date, there is only one drug approved by the Food and Drug Administration (FDA) for the treatment of Covid-19 patients.

Remdesivir, a polymerase inhibitor initially conceived to target Ebola Virus, proved to be efficient in shortening the recovery time in adult patients hospitalised with Covid-19^5^^,^^6^ and has therefore been granted Emergency Use Authorisation (EUA). Unfortunately, due to its pharmacokinetic profile, this drug has to be administered intravenously in a hospital setting, thereby limiting its use for Covid-19 treatment on a massive scale. The first attempts to face this lack of pharmacological tools to contrast the Covid-19 pandemic involved the repurposing of antiviral drugs designed for the treatment of other virus-related illnesses against Covid-19: this approach, despite being very appealing from a timescale perspective^7^, did not bring any significant results, with several clinical trials showing little to no efficacy of those active principles against SARS-CoV-2^8^.

Meanwhile, the early release to the scientific community of the crystallographic structure of the SARS-CoV-2 main protease (M^pro^) (PDB ID: 6LU7), caused a shift in the attention of researchers around the world towards the Structure-Based approach to the rational design of new potential protease inhibitors^9^^,^^10^. Among all the different chemical entities developed to target the main protease, PF-07321332 is, to date, the first and only orally available COVID-19 antiviral clinical candidate.

Designed amid the pandemic, the structure of PF-07321332 was unveiled by Pfizer on April 6th at the American Chemical Society Spring 2021 meeting^11^. This compound, which has recently entered clinical phase I, was developed to target SARS-CoV-2 main protease, thereby impairing the virus's ability to reproduce itself, and it is intended as a pharmacological tool to prevent the development of COVID-19 in people who have been exposed to the pathogen. Even though the compound structure has been revealed, no further information has been provided yet about the way PF-07321332 interacts with the main protease active site, except for the fact that it reacts reversibly with a cysteine residue located in the binding site^11^.

In this perspective computational investigation, we exploited Supervised Molecular Dynamics (SuMD)^12^, an emerging protocol allowing to decipher at an atomic level of detail the recognition process between two molecular entities, to sample and characterise a putative binding pathway for PF-07321332. As described in the original publication, SuMD simulations fully consider both the protein flexibility and the contribution of the solvent molecules, which are explicitly simulated, throughout the binding process. As shown by previous scientific works^13^^,^^14^, this makes it possible to overcome the limitations of traditional techniques such as molecular docking when working on challenging targets such as M^pro^, whose active site is relatively shallow, plastic and solvent exposed^15^.

## Methods

### Software overview

For every general molecular modelling operation, such as protein and ligand structure preparation, MOE suite (Molecular Operating Environment, version 2019.01^16^) was used, exploiting an 8 CPU (Intel Xeon E5-1620 3.50 GHz) Linux Workstation. Molecular Dynamics simulations were carried out with ACEMD^17^ (version 3.3.0), which is based upon OpenMM^18^ (version 7.4.0), on a cluster composed of 20 NVIDIA GPUs.

### Structure preparation

The crystallographic structure of the unliganded M^pro^ was retrieved from the Protein Data Bank (PDB ID: 7K3T). At first, the active functional dimer of the protease was restored applying the symmetric crystallographic transformation to each asymmetric unit. Residues with alternative conformation were assigned to the one with the highest occupancy. The Protonate3D tool was then used to add missing hydrogen atoms, evaluating the most probable protonation state for each titratable residue at pH 7.4. Finally, each non-protein residues (e.g.: water, co-solvents, etc.) were removed before successive steps. The ligand structure was prepared exploiting tautomers, fixpka, and molcharge tools from the QUACPAC OpenEye^19^ software suite to assign the most probable tautomeric and protomeric state at pH 7 and ligand partial charges according to the MMFF94 force field. Three-dimensional coordinates were generated with Corina Classic^20^.

### Molecular dynamics system setup

The simulated system, composed by 119979 atoms, contained both the protein and the ligand structure prepared as described in the previous section, with the ligand positioned at least 30 Å away from the nearest receptor atoms. For system parametrization, the combination of Amber ff14SB and General Amber Force Field (GAFF) was used to describe each component of the simulation box.

The system was explicitly solvated in a cubic TIP3P^21^ water box with 15 Å padding and neutralised with the addition of Na^+^/Cl^-^ ions until a 0.154 M concentration was reached. Prior to the simulation, 1000 steps of energy minimisation with the conjugated-gradient algorithm were performed. A two-step equilibration stage was carried out in the following way: the first step consisted of 0.1 ns of simulation in the canonical ensemble (NVT) with harmonic positional restraints applied both on the protease and ligand atoms using a 5 Kcal mol^−1 ^Å^−2^ force constant, the second step consisted of 0.5 ns of simulation in the isothermal-isobaric ensemble (NPT) with the same harmonic positional restraints applied only on protein alpha carbons and ligand atoms. For each simulation, an integration timestep of 2 fs was used. To constrain bonds involving hydrogen atoms the M-SHAKE algorithm was used. A 9.0 Å cut-off was applied for the calculation of Lennard-Jones interactions, while electrostatic interactions were computed exploiting the particle-mesh Ewald method (PME). The temperature was maintained at the constant value of 310 K by the Langevin thermostat, with a friction coefficient of 0.1 ps^−1^. During the second equilibration stage, the pressure was maintained constant at 1.0 atm utilising a Monte Carlo barostat.

### Supervised Molecular Dynamics (SuMD) simulation

SuMD code is written in Python 2.7 and exploits the ProDy^22^ package to perform geometrical supervision upon the ligand-binding process. This supervision allows to reduce the timescale, hence shrinking the computational effort, that is required to sample the ligand-biomolecular target recognition process to the range of nanoseconds, instead of the usual hundreds of nanoseconds or microseconds that are required by unbiased molecular dynamics (MD) simulations. The entire SuMD derived trajectory is composed by short unbiased 600 ps MD simulation runs (NVT ensemble, T = 310 K) carried out with the ACEMD3 software: at the end of each simulation (the so-called “SuMD-step”), the distance between the centre of mass of the ligand and the binding site is computed at five different points, picked at regular time intervals, and fitted into a linear function evaluated by a tabu-like algorithm. Only those SuMD-steps whose computed slope is negative (indicating that the ligand is approaching the binding site) are retained. Every time a SuMD-step is rejected (positive slope), the simulation is restarted from the previous productive step by randomly assigning the atomic velocities. The supervision algorithm is switched off after the distance between the centre of mass of the ligand and the binding site drops below 5 Å: from that point on the simulation continues as a classical MD simulation.

## Results

In our computational study, we exploited Supervised Molecular Dynamics simulations to obtain a putative binding pathway between PF-07321332 and the SARS-CoV-2 Main Protease (M^pro^) catalytic site. A total amount of 36 ns of SuMD simulation time proved sufficient to sample the entire recognition trajectory, from the starting unbound state to the final predicted protein-ligand complex.

As can be seen in Video 1 (Supplementary Material), PF-07321332 reaches M^pro^ active site after about 7 ns of simulation time, making its first contacts with Leu141, Asp 142, Gln189, and Glu166. Leu141 and Asp142 are part of the oxyanion loop (residues 138–145), which lines the binding pocket of Glutamine P1 and is assumed to stabilise the tetrahedral acyl transition state^15^. Glu166 is a key residue located in the middle of the binding site: mutagenesis studies carried out on SARS-CoV M^pro^ (which has 96% sequence identity with SARS-CoV-2 M^pro^ and is identical at the binding site level^13^) showed that this residue plays a key role in linking the dimer interface with the substrate-binding site^23^. Gln189 is located at the boundary of the S3 site and is assumed to be one of the key interactors with SARS-CoV-2 M^pro^ inhibitors, as well as Glu166^24^. Asn142 and Gln189, located on opposite sides at the boundary of the binding sites, seem to serve as electrostatic recruiters for the ligand, exploiting their polar and flexible sidechains to manoeuvre the entrance of the ligand into the core region of the binding site. Glu166 appears to instead serve as an electrostatic anchor that tightly hooks the middle portion of the ligand with the central region of the binding site, facilitating the formation of further interactions with residues such as His164.

After the tri-fluoro-acetamide moiety of the compound establishes contact with the side chain of Gln189, the cyclopropyl-proline moiety occupies the central portion of the binding site, establishing a series of coordinated hydrogen bonds with the backbone of His164 and Glu166 and orientating the cyclopropyl group towards the hydrophobic S2 pocket, delimited by the side chains of His41, Met49, Tyr54, and Met165. Meanwhile, the pyrrolidone moiety is inserted in the S1 pocket, interacting with key residues of the oxyanion loop such as Asn142, Gly143, and Ser144, before undergoing a conformational rearrangement around the 18 ns simulation time mark which allows the carbonyl of the pyrrolidone to establish a hydrogen bond with His163. This interaction has been flagged as a conserved interaction across several deposited structures of non-covalent inhibitors^25^. Moreover, this interaction is conserved across all possible substrate peptide crystal structures, where the interacting group is the sidechain of the Glutamine P1 residue^26^.

Subsequently, the pyrrolidone moiety rearrangement also allows the reactive nitrile group to face the catalytic Cys145, making it possible to reach the final covalent-bound state which cannot be described through molecular mechanics. Finally, in the final conformation, the tri-fluoro acetamide moiety is fully inserted in the S4 subpocket, establishing two additional hydrogen bonds with the backbone of Thr190 and Glu166.

**Figure 1.**
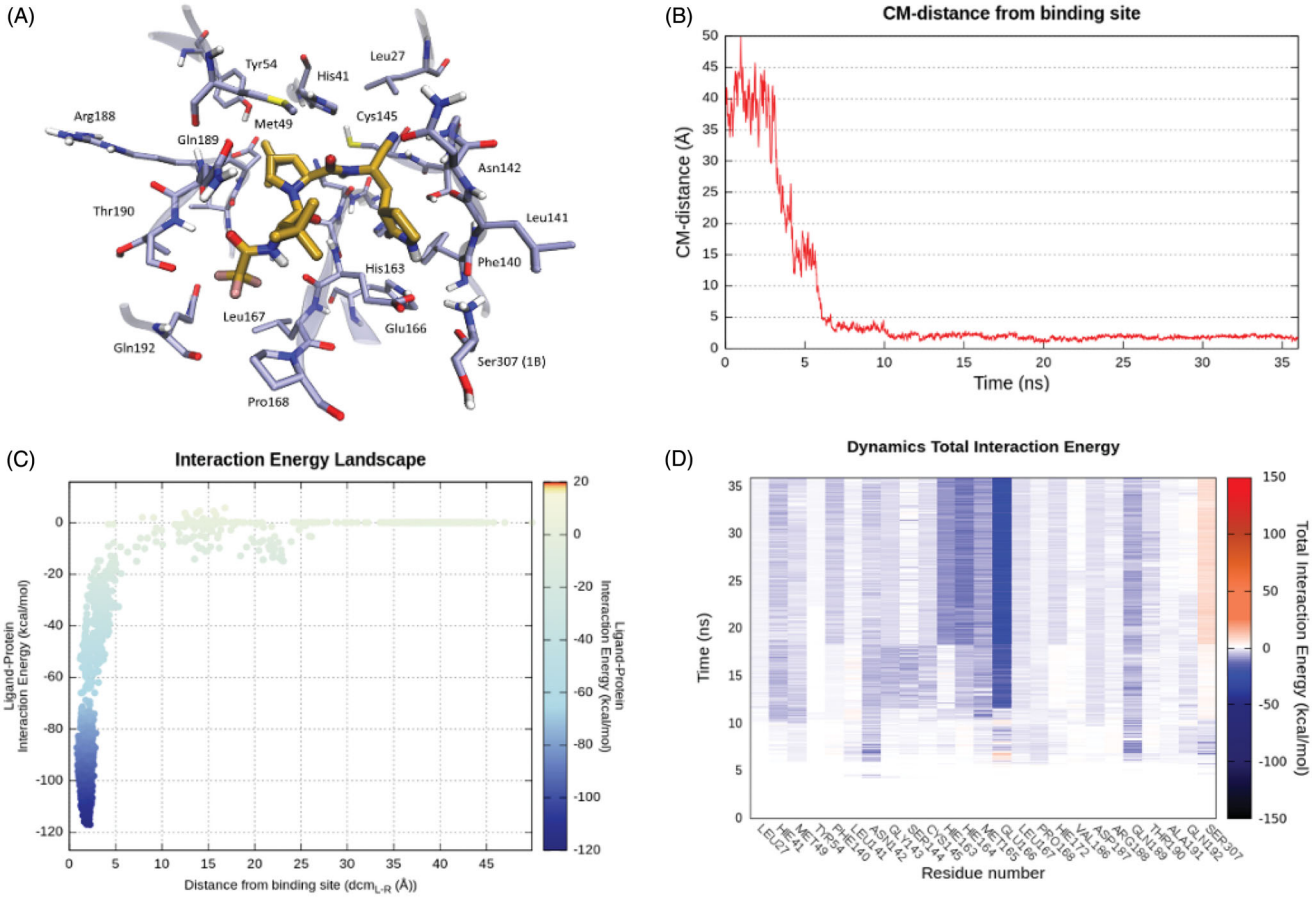
This panel encompasses the recognition pathway between PF-07321332 and the SARS-CoV-2 main protease predicted by SuMD. (A) PF-07321332 conformation within the binding site, sampled in the last SuMD trajectory frame (orange). Binding site residues within 4 Å of the ligand are depicted in ice-blue. (B) Profile of the distance between the centre of mass of the ligand and the M^pro^ catalytic site during SuMD simulation. (C) Interaction Energy Landscape describing the protein-ligand binding pathway; values are arranged according to distances between the centre of mass of the ligand the one of the M^pro^ catalytic site. (D) Dynamic total interaction energy (sum of electrostatic and van der Waals contribution) computed for the 25 most contacted residues throughout the SuMD trajectory.

As can be seen in Figure 1 (Supplementary Material), the ligand conformation in the final step of the SuMD simulation is superimposable to the bound state predicted by the PLANTS^27–29^ docking algorithm (RMSD_SuMD-PLANTS_: 0.92 Å), further corroborating the binding mode hypothesis portrayed by the SuMD protocol.

## Discussion

**Figure 2.**
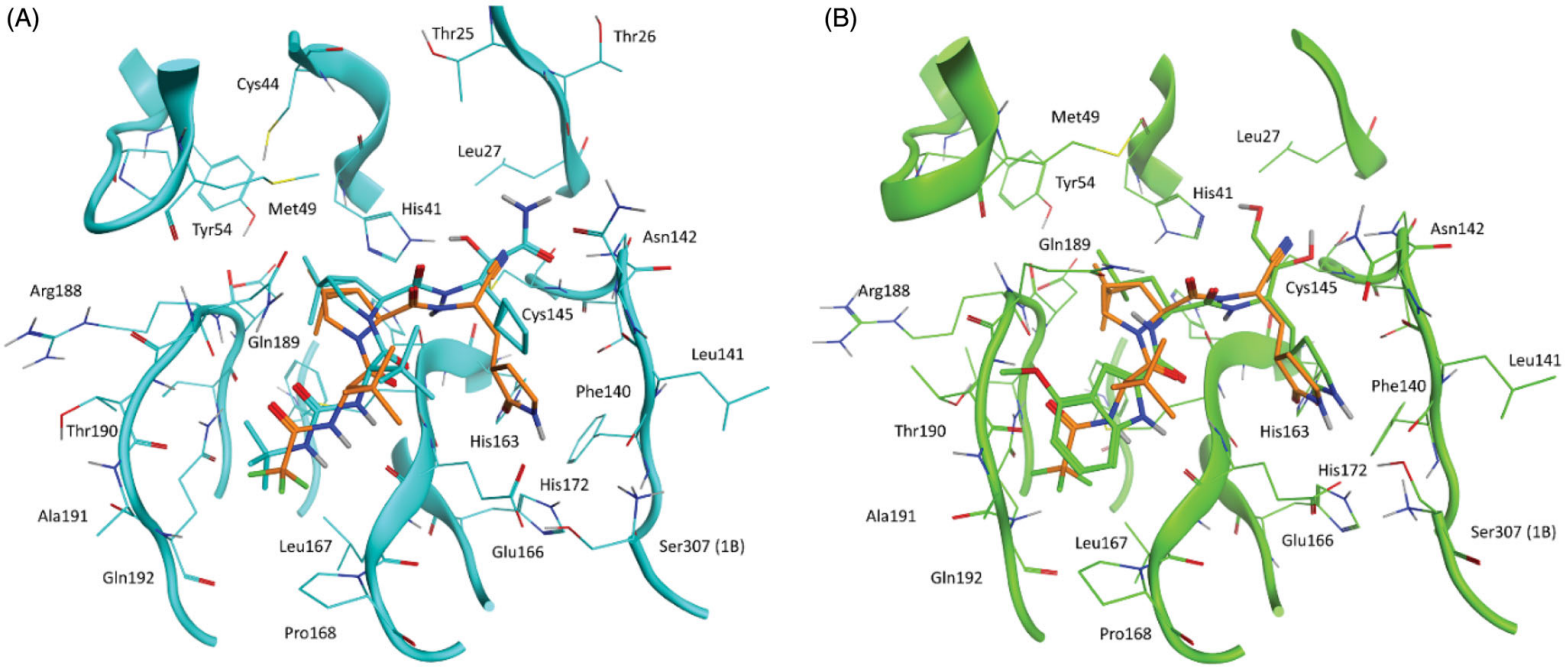
This panel illustrates the similarities between PF-07321332 conformation in the final SuMD trajectory frame and the crystallographic complexes of two structurally related covalent inhibitors of SARS-CoV-2 M^pro^: Boceprevir and PF-00835231 (active metabolite of PF-07304814). (A) superposition between the binding mode predicted by SuMD for PF-07321332 (orange) and the crystallographic complex of Boceprevir within the catalytic site of SARS-CoV-2 M^pro^ (cyan, PDB ID: 6WNP). (B) superposition between the binding mode predicted by SuMD for PF-07321332 (orange) and the crystallographic complex of PF-00835231 within the catalytic site of SARS-CoV-2 M^pro^ (green, PDB ID: 6XHM).

Intriguingly, the binding mode proposed by the SuMD simulation for PF-07321332 is fairly superimposable to the ones of other two covalent protease inhibitor, Boceprevir (PDB ID: 6WNP) and PF-00835231 (PDB ID: 6XHM), which share common structural features with the oral candidate, validating the hypothesis that they could also share an overall similar interacting pattern (Figure 2).

Boceprevir is a protease inhibitor originally developed for the Hepatitis C Virus (HCV) NS3 protease^30^. It shares many common structural features with PF-07321332, such as the cyclopropyl proline residue at P2 and the alanine at the P3 position but has a different reactive group (α-ketoamide), a cyclobutyl alanine at P1, and a tert-butyl carbamate capping moiety at P4. From a binding mode point of view, the most prominent difference between the newly developed inhibitor and Boceprevir regards the hydrogen bond with His163 (absent in Boceprevir complex with the protease) which, as previously mentioned, is a crucial interaction also for natural peptidic substrates.

PF-07304814 is a Phase I clinical candidate originally developed by Pfizer in 2002–2003 against SARS-CoV and repurposed for SARS-CoV-2 due to the aforementioned similarities between the two viruses proteases^31^. The compound contains a hydrolysable phosphate group which enhances its solubility and is cleaved by alkaline phosphatases in tissue releasing the active compound PF-00835231. The main limiting factor for this candidate is that, unlike its successor PF-07321332, it has to be administered intravenously, making it less appealing for massive distribution and relegating its usage to hospital settings. From a structural point of view, this latter compound is less similar to PF-07321332 compared to Boceprevir, but still retains the key features concerning its binding mode with the MPro active site. The only conserved structural feature between the two inhibitors developed by Pfizer is the pyrrolidone group at the P1 position, which establishes a hydrogen bond with His163. The reactive group, in this case, is an aldehyde, the same as for Boceprevir. The hydrophobic residue at P2, in this case, is a leucine, which is the most recurrent amino acid that can be found at the P2 position in natural substrate peptides (included the N-term of M^pro^ itself)^26^, while the P3 terminal residue is a 4-methoxyl indole group, which interacts through a hydrogen bond with the backbone of Glu166. Additional interaction occurs at the P1’ subsite, where the two hydroxyl groups (one of which is formed upon reaction between the aldehyde group and Cys145 sidechain) form hydrogen bonds with Cys145 backbone and His41 sidechain.

Overall, PF-07321332 appears to have combined the strong points of both Boceprevir and PF-07304814 in a single molecular entity, showing that it is possible to repurpose the knowledge acquired in previous drug development campaigns on different virus proteases to rationally design SARS-CoV-2 M^pro^ inhibitors suitable for advancement to clinical phases, hence addressing the need for a quick response against a widespread disease like Covid-19. Moreover, the combination of innovative computational strategies such as SuMD with experimental data coming from X-Ray Crystallography could provide useful structural insights to stir the rational development of antiviral drugs in a more rational and less time-consuming way.

## Conclusions

In this computational study, we employed Supervised Molecular Dynamics (SuMD) to investigate the recognition process between PF-07321332, the first orally available Covid-19 antiviral candidate to reach clinical phase I, and its biological target, SARS-CoV-2 main protease (M^pro^).

About 36 ns of SuMD simulations proved sufficient to sample a putative binding process, allowing to simulate the whole approaching path from the unbound state to the final protein-ligand complex. SuMD simulations suggest a possible role in the first stages of the recruitment of the ligand for residues such as Leu141, Asp 142, Gln189, and Glu166, which have already been acknowledged as crucial residues for the binding of both natural and synthetic substrates.

Finally, the binding mode predicted by SuMD for PF-07321332 is quite similar for other structurally related protease inhibitors, namely Boceprevir and PF-07304814, which could also share a similar binding pathway.

## Supplementary Material

Supplemental Material
